# Cardiac effects in perinatally HIV-infected and HIV-exposed but uninfected children and adolescents: a view from the United States of America

**DOI:** 10.7448/IAS.16.1.18597

**Published:** 2013-06-18

**Authors:** Steven E Lipshultz, Tracie L Miller, James D Wilkinson, Gwendolyn B Scott, Gabriel Somarriba, Thomas R Cochran, Stacy D Fisher

**Affiliations:** 1 Department of Pediatrics, Jackson Memorial Medical Center and the Sylvester Comprehensive Cancer Center, Holtz Children's Hospital, University of Miami Miller School of Medicine, Miami, FL, USA; 2 Departments of Medicine and Pediatrics, Comprehensive Heart Center, University of Maryland School of Medicine, Baltimore, MD, USA

**Keywords:** HIV, AIDS, child, cardiac outcomes, antiretroviral therapies, therapeutic complications, cardiovascular risk

## Abstract

**Introduction:**

Human immunodeficiency virus (HIV) infection is a primary cause of acquired heart disease, particularly of accelerated atherosclerosis, symptomatic heart failure, and pulmonary arterial hypertension. Cardiac complications often occur in late-stage HIV infections as prolonged viral infection is becoming more relevant as longevity improves. Thus, multi-agent HIV therapies that help sustain life may also increase the risk of cardiovascular events and accelerated atherosclerosis.

**Discussion:**

Before highly active antiretroviral therapy (HAART), the two-to-five-year incidence of symptomatic heart failure ranged from 4 to 28% in HIV patients. Patients both before and after HAART also frequently have asymptomatic abnormalities in cardiovascular structure. Echocardiographic measurements indicate left ventricular (LV) systolic dysfunction in 18%, LV hypertrophy in 6.5%, and left atrial dilation in 40% of patients followed on HAART therapy. Diastolic dysfunction is also common in long-term survivors of HIV infection. Accelerated atherosclerosis has been found in HIV-infected young adults and children without traditional coronary risk factors. Infective endocarditis, although rare in children, has high mortality in late-stage AIDS patients with poor nutritional status and severely compromised immune systems. Although lymphomas have been found in HIV-infected children, the incidence is low and cardiac malignancy is rare. Rates of congenital cardiovascular malformations range from 5.6 to 8.9% in cohorts of HIV-uninfected and HIV-infected children with HIV-infected mothers. In non-HIV-infected infants born to HIV-infected mothers, foetal exposure to ART is associated with reduced LV dimension, LV mass, and septal wall thickness and with higher LV fractional shortening and contractility during the first two years of life.

**Conclusions:**

Routine, systematic, and comprehensive cardiac evaluation, including a thorough history and directed laboratory assays, is essential for the care of HIV-infected adults and children as cardiovascular illness has become a part of care for long-term survivors of HIV infection. The history should include traditional risk factors for atherosclerosis, prior opportunistic infections, environmental exposures, and therapeutic and illicit drug use. Laboratory tests should include a lipid profile, fasting glucose, and HIV viral load. Asymptomatic cardiac disease related to HIV can be fatal, and secondary effects of HIV infection often disguise cardiac symptoms, so systematic echocardiographic monitoring is warranted.

## Introduction

(HIV) infection is a primary cause of acquired heart disease, particularly of accelerated atherosclerosis, symptomatic heart failure, and pulmonary arterial hypertension [1–17]. Cardiac complications often occur in the later stages of HIV infection with prolonged viral infection and are therefore becoming more relevant as longevity improves [1–17]. Multi-agent HIV therapies that help sustain life may also directly increase the risk of cardiovascular events and accelerated atherosclerosis [1,18–22].

By 2011, between 31 and 36 million people were living with HIV [23], an estimated 0.8% of all people aged 15–49 years. Globally, treatment and burden of this epidemic varies greatly from one region to the next. One of the most severely afflicted regions is sub-Saharan Africa, where 69% of all people living with HIV reside and nearly 1 in every 20 adults is infected [23]. In 2011, 330,000 children acquired HIV infection (90% of whom are in sub-Saharan Africa), 43% less than in 2003 [23].

HIV-infected children did not usually receive antiretroviral therapy (ART) or only received monotherapy with zidovudine in the early 1990s. These children often experienced abnormal left ventricular (LV) structure and function, a predictor of mortality [24]. Although the cardiovascular effects of HIV and ART are not fully understood, HIV-infected children are routinely exposed to ART or highly active antiretroviral therapy (HAART) while the cardiovascular system is still developing. Sub-clinical cardiac abnormalities may develop into symptomatic cardiomyopathy in adulthood.

Cardiac abnormalities (Table 1) associated with HIV infection include premature myocardial infarction (MI) or stroke, pericardial effusion, lymphocytic interstitial myocarditis, LV diastolic dysfunction, dilated cardiomyopathy (frequently with myocarditis) infective endocarditis, and malignancy (myocardial Kaposi's sarcoma and B-cell immunoblastic lymphoma; Table 1) [3,4,25,26]. Treatment-related drug effects and interactions are considerably more prevalent and directly challenge the cardiovascular system through lipid abnormalities with protease inhibitors (PIs) and an increased statin serum concentration with PIs [18]. Therapies may also change repolarization or prolong the QT interval, increasing the risk of sudden cardiac death [18].

**Table 1:** Summary of HIV-associated cardiovascular diseases

Disease	Possible causes	Incidence/prevalence	Diagnosis	Treatment
Accelerated atherosclerosis	Protease inhibitors, atherogenesis with virus-infected macrophages, chronic inflammation, glucose intolerance, dyslipidemia, endothelial dysfunction	Up to 8% prevalence	ECG, Stress testing, echocardiography, lipid profile, CT angiography, and calcium scoring	Smoking cessation, low fat diet, aerobic exercise, blood pressure control, guideline based statin use, percutaneous coronary intervention, coronary artery bypass surgery
Dilated cardiomyopathy systolic dysfunction	*Coronary Artery Disease Drug related:* cocaine, AZT, IL-2, doxorubicin, interferon *Infectious:* HIV, toxo-plasma, coxsackievirus group B, EBV, CMV, adenovirus *Metabolic or endocrine:* Selenium or carnitine deficiency, anaemia, hypocalcemia, hypophosphatemia, hyponatremia, hypokalemia, hypoalbuminemia, hypothyroidism, growth hormone deficiency, adrenal insufficiency, hyperinsulinemia *Cytokines:* TNF-α, nitric oxide, TGF-β, endothelin-I, interleukins *Immunodeficiency:* CD4 <100 *Autoimmune*	Up to 8% of asymptomatic patients Up to 25% of autopsy cases	*Chest radiograph findings* *ECG: Nonspecific conduction abnormalities,* PVCs, PACs *Echocardiogram findings:* low-normal LV wall thickness, increased LV mass, dilated LV, systolic LV dysfunction. *Possible laboratory studies:* Troponin T, brain natriuretic peptide level, CD4 count, viral load, viral PCR, toxoplasma serology, thyroid-stimulating hormone, cortisol, carnitine, selenium, serum ACE, stress testing, myocardial biopsy, cardiac catheterization	Diuretics, digoxin, ACE inhibitors, β-blockers Adjunctive treatment in HIV patients Treatment of infection nutritional replacement IVIg Intensify antiretroviral therapy Follow-up serial echocardiograms
LV diastolic dysfunction	*TNF, Interleukin (6)* *Hypertension* *Chronic viral infection*	Up to 37% asymptomatic	Echocardiography Tissue doppler imaging	Treat hypertension Intensify antiretroviral therapy
Pulmonary hypertension	Plexogenic pulmonary arteriopathy	0.5%	ECG, echocardiography, right heart catheterization	Anticoagulation, vasodilators, prostacyclin analogs Endothelin antagonists, PDE-5 Inhibitors
Pericardial disease	*Bacteria: Staphylococcus*, *Streptococcus*, *Proteus*, *Klebsiella*, Pericardiocentesis *Enterococcus*, *Listeria*, *Nocardia*, *Mycobacterium Viral pathogens:* HIV, HSV, CMV, adenovirus, echovirus *Other pathogens:* *Cryptococcus*, *Toxoplasma*, *Histoplasma* *Malignancy:* Kaposi’s sarcoma, lymphoma, capillary leak/wasting/malnutrition *Hypothyroidism* *Immunodeficiency* *Uremia*	11%/year- markedly reduced in post HAART studies. Spontaneous resolution in 42% of affected patients Approximately 30% increase in six-month mortality	Pericardial rub on examination Echocardiogram Fluid analysis for gram stain, and culture, cytology ECG-low voltage/PR depression Associated pleural and peritoneal fluid analysis Pericardial biopsy	Treat the cause *Followup:* Serial echocardiograms Intensify antiretroviral therapy Pericardiocentesis or window *Histoplasma*
Infective endocarditis	*Autoimmune Bacteria: Staphylococcus aureus* or *Staphylococcus epidermidis*, *Salmonella*, *Streptococcus*, *Hemophilus parainfluenzae*, *Pseudallescheria boydii*, *HASEK organisms* *Fungal:* *Aspergillus fumigatus, Candida, Cryptococcus neoformans*	Increased incidence in IVDA, regardless of HIV status	Blood cultures; Echocardiogram	IV antibiotics, valve replacements
Nonbacterial thrombotic endocarditis	Valvular damage, vitamin C deficiency, malnutrition, wasting, DIC, hypercoagulable state, prolonged acquired immunodeficiency	Rare condition, but clinically relevant emboli in 42% of cases	Echocardiogram	Anticoagulation, treat vasculitis or underlying illness
Malignancy	Kaposi’s sarcoma, non-Hodgkin lymphoma, leiomyosarcoma Low CD4 count, prolonged immunodeficiency HHV-8, EBV	Approximately 1% incidence Usually metastatic in HIV+ patients	Echocardiogram, biopsy	Chemotherapy possible
Right ventricle disease	Recurrent pulmonary infections, pulmonary arteritis, microvascular pulmonary emboli, COPD		ECG, echocardiography, right heart catheterization	Diuretics, treat underlying lung infection or disease, anticoagulation as clinically indicated
Vasculitis	Drug therapy with antibiotics and antivirals	Increasing incidence	Clinical diagnosis	Systemic corticosteroids, withdrawal of drug
Autonomic dysfunction	CNS disease, drug therapy, prolonged immunodeficiency, malnutrition, sedentary lifestyle	Increased in patients, with CNS disease	Tilt-table test, Holter or Event monitoring	Procedural precautions
Arrhythmias	Drug therapy, pentamidine, autonomic dysfunction, acidosis electrolyte abnormalities		ECG—long QT, Holter monitoring, exercise stress testing	Discontinue drug, procedural precautions Electrolyte replacement
Lipodystrophy	*Drug therapy:* protease inhibitors		Echocardiography, lipid profile, cardiac catheterization, coronary calcium score	Lipid therapy (beware of drug interactions), aerobic exercise, altered antiretroviral, therapy, cosmetic surgery/fat implantation

ACE=angiotensin-converting enzyme; AZT=azidothymidine; CMV=cytomegalovirus; CNS=central nervous system; DIC=disseminated intravascular coagulation; EBV=Epstein-Barr virus; ECG=electrocardiogram; HHV=human herpes virus; HIV=human immunodeficiency virus; HSV=herpes simplex virus; HTN=hypertension; IL-2=interleukin-2; IVDA=intravenous drug abuse; IVIg=intravenous immunoglobulin; LV=left ventricular; PAC=premature atrial complex; PCR=polymerase chain reaction; PVC=premature ventricular complex; TGF=transforming growth factor; TNF=tumour necrosis factor. Modified with permission from Fisher SD, Lipshultz SE. Chapter 72: Cardiovascular abnormalities in HIV-infected individuals. In: Braunwald's Heart Disease: A Textbook of Cardiovascular Medicine, Ninth Edition. Editors: Bonow RO, Mann DL, Zipes DP, Libby P. Philadelphia: Elsevier Saunders. 1618–27. 2011 ISBN: 978-1-4377-0398-6.

## Discussion

### Accelerated atherosclerosis

Since the advent of ART, patients with HIV infection have longer life expectancies, but chronic conditions including atherosclerotic and metabolic disease are becoming more prevalent in this population [27]. Highly active ART (HAART) causes a metabolic syndrome well-characterized in adults as unfavourable body composition (reduction in subcutaneous and increase in visceral fat), insulin resistance and abnormal glucose metabolism, and dyslipidemia [28,29]. The physiologic effect of the metabolic syndrome places patients at risk for atherosclerotic cardiovascular disorders. In fact, in adults with HAART-related fat redistribution, several studies have suggested an increase in the risk of MI relating to the level of viral control (increased inflammation) or to ART exposures (including PIs and certain nucleoside reverse transcriptase inhibitors) [30–32]. Acute MI can be the primary presentation of atherosclerotic disease [33]. However, there is controversy over whether the metabolic syndrome in HIV-infected patients is exclusively related to ART exposure or HIV infection itself. Synergistic causes may include traditional risk factors such as family history, high LDL cholesterol, low HDL cholesterol, diabetes, hypertension, age >55, HIV viral load, and medication specific ART exposure. Studies in children show similar although not identical findings, including abnormal body composition, insulin resistance, and dyslipidemia with the use of ART, with increased risk at older age and longer duration of HAART [34–40]. The onset of puberty has been proposed as another factor that is associated with accelerating these changes [41].

Early studies in children showed that PI therapy improved weight, weight-for-height and mid-arm muscle circumference of HIV-infected children, independent of the concurrent decrease in HIV viral load and improved CD4 T-lymphocyte counts [42]. The immediate treatment effects were most apparent with an improvement in weight and mid-arm muscle circumference and there was a trend towards increased height and lean body mass. In addition to the positive improvements in growth and lean body mass, however, HAART is also associated with abnormalities in fat distribution in children though some studies report similar lean mass in HIV-infected and uninfected children [43]. Arpadi *et al*. observed similar total fat, trunk fat, and percentage of total fat between HIV-infected and uninfected children, but lower leg and higher arm fat in infected children [44]. Jacobson *et al*. showed there were decreased limb/trunk fat ratios in HIV-infected children when compared with HIV-exposed uninfected (HIVEU) [35]. These findings suggest that both peripheral lipoatrophy, as well as central obesity occur in these children. Further studies have shown that a majority of children develop fat redistribution within three years of initiating a protease inhibitor (PI) - containing regimen, and that these changes progress over time [45]. Other studies have identified metabolic abnormalities induced by other specific classes of drugs. Stavudine use has been associated with lipoatrophy [46], potentially by altering mitochondrial number and function [47].

Following exposure to ART, there are increases in total, LDL, and HDL cholesterol in both adults and children. Children newly exposed to ART experienced a rapid rise in LDL cholesterol over the first six months that continued through 12 months [48]. A total of 10% of a cohort of 449 children in the United Kingdom had LDL cholesterols over the 95% for age and PIs caused greater rises in total cholesterol than non-nucleoside reverse transcriptase inhibitors. The authors concluded that dietary and exercise interventions and a change in ART might help address these metabolic abnormalities [49]. In children with incident hypercholesterolemia, Jacobson *et al*. found that a switch in the ART regimen was associated with cholesterol levels that returned to normal [50]. There was limited power to detect the effects of switching to specific ARTs; however, a higher viral load at baseline was associated with the normalization of cholesterol. According to the Department of Health and Human Services Panel on Antiretroviral Guidelines for Adults and Adolescents, switching from one PI to another PI or to the same PI at a lower dosing frequency may reduce dyslipidemia [51]. Evaluating metabolic changes in children as they start or change ART can be helpful to determine specific effects of ART because children have fewer confounding psychosocial factors (such as smoking, alcohol, obesity) that can independently impact metabolic outcomes.

**Figure 1:**
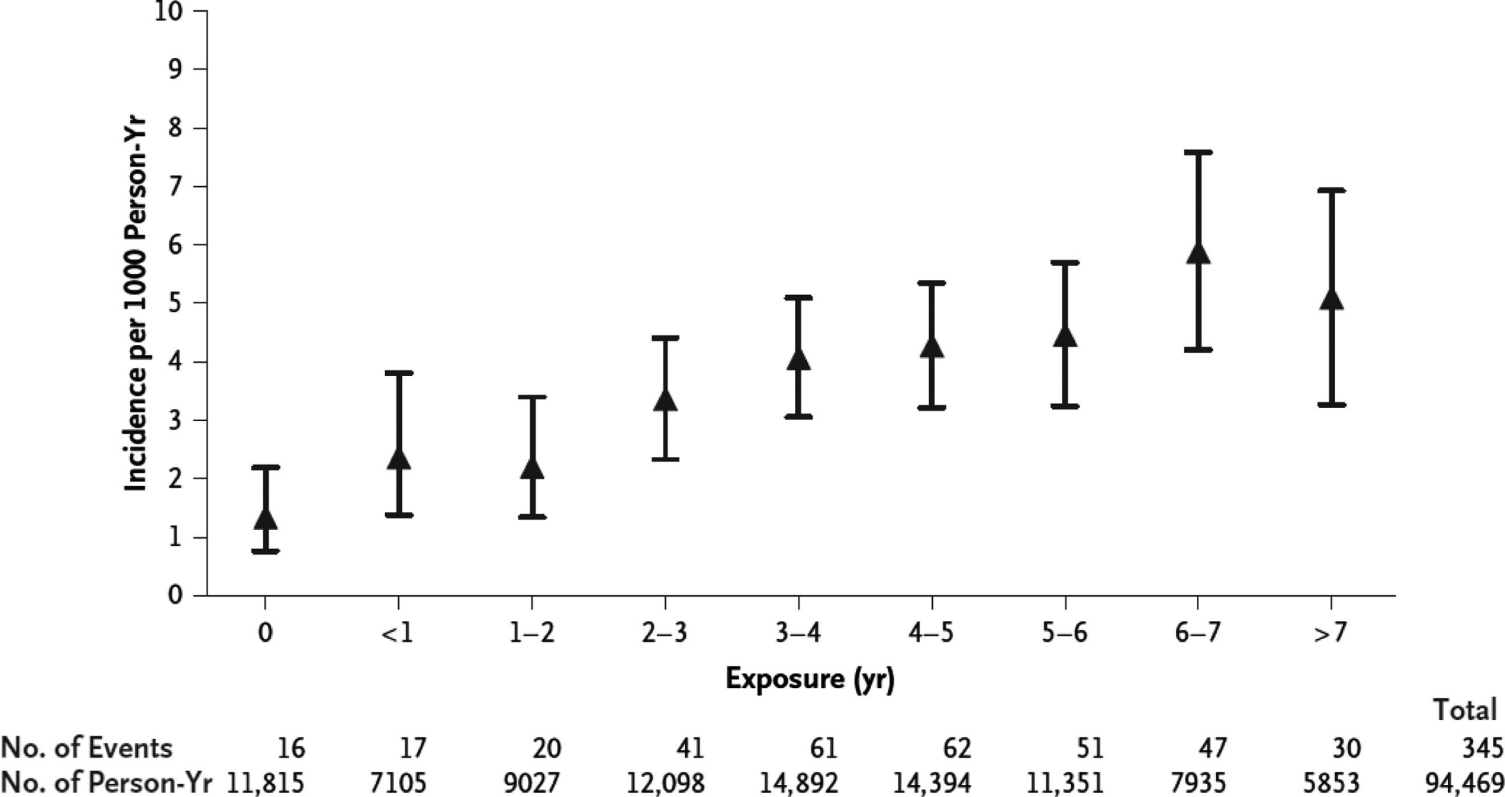
Risk of myocardial infarction according to exposure to combination antiretroviral therapy. The adjusted relative rate of myocardial infarction according to cumulative exposure to combination antiretroviral therapy was 1.16 per year of exposure (95% CI, 1.09–1.23). The I bars denote the 95% CIs. Reproduced with permission from ref. 57.

Atherosclerotic cardiovascular disease (CVD) often results from an environment that is hostile to the endothelium, which may occur from a complex interaction of HIV, the adverse effects of ART, traditional risk factors for CVD, inflammation and co-infections [52]. Autopsies in HIV-infected patients aged 23–32 years who died unexpectedly revealed atherosclerotic plaque with features common to both coronary atherosclerosis and transplant vasculopathy, histologic characteristics more frequently seen with single-vessel disease in which the cause of MI is plaque rupture [53,54]. Imaging data suggest inflammation as the cause of such premature cardiovascular events (Table 2). Endothelial dysfunction is one possible causative link between HIV infection and atherosclerosis. HIV-infected patients have increased expression of vascular adhesion molecules (E-selectin, ICAM, VCAM) and inflammatory cytokines such as interleukin (IL-6) and tumour necrosis factor (TNF)-α [55,56]. The presence of an endothelial response to injury is supported by the correlation of viral load with higher plasma TNF-α, IL-6, and von Willebrand factor concentrations [37,55,57]. The risk of myocardial infarction has been found to increase with the exposure to combination ART (Figure 1) [57].

**Table 2:** Imaging and support that atherosclerosis is inflammatory in HIV-infected people

Modality	HIV vs Matched Controls	Associations
Carotid ultrasound Carotid intimal-medial thickness	First to show higher rates of atherosclerosis 0.04 mm thicker in HIV (metanalysis)	Smoking, dyslipidemia, low nadir CD4 T-cell count, and increased lymphocyte activation correlated with higher IMT and progression
Computed tomography calcium scores	HIV-infected have higher mean Agatston scores and proportion of scores >0	Framingham risk, metabolic syndrome, higher levels of asymmetric dimethylarginine, and fatty liver
CT angiography	Higher prevalence of noncalcified plaque	CD4/CD8 ratio and HIV duration independently predict plaque burden
Magnetic resonance angiography		Association of HIV viremia and atherosclerotic plaque burden in the aorta Extensively used in cerebral and peripheral vascular beds
Flow-mediated brachial artery dilation	Impaired in HIV-infected	Degree of HIV viremia, injection drug use, periodontal disease, and vitamin D deficiency Statins, niacin, and pentoxifylline have been beneficial in improving flow-mediated dilation
Future potential imaging Intravascular ultrasound Intracoronary optical coherence tomography Future PET imaging of 18 FDG uptake Molecular targeted magnetic resonance imaging		

Modified with permission from Fisher SD, Lipshultz SE. Chapter 72: Cardiovascular abnormalities in HIV-infected individuals. In: Braunwald's Heart Disease: A Textbook of Cardiovascular Medicine, Ninth Edition. Editors: Bonow RO, Mann DL, Zipes DP, Libby P. Philadelphia: Elsevier Saunders. 1618–27. 2011 ISBN: 978-1-4377-0398-6.

Premature cerebrovascular disease is also prevalent in HIV-infected adults and providers should be aware of its risks in young adults. A review of autopsies from 1983 to 1987 found that AIDS patients had an estimated 8% prevalence of stroke. Evidence of cerebral emboli was found in four of the 13 patients with stroke and the embolus had a clear cardiac source in three of these four patients. Considering these patients were in the pre-HAART era, the aetiology is possibly different. As HIV-infected children age, the common origins of stroke should be sought and atherosclerotic disease should be suspected. Premature atherosclerosis is generally found in children treated with ART, although it is not clearly ART related. Acute stroke investigation in HIV-infected individuals should be somewhat different than in the general population as a result of infectious and immune-mediated vasculopathy, tumours, opportunistic infections, and cardioembolism [58].

Prevention of premature CVD should be directed at identifying and decreasing known risk factors. Low cholesterol diets reduce the incidence of dyslipidemia [59]. In addition, patients should be encouraged to follow heart-healthy diets with increased aerobic activities and avoid smoking as it has been found that exercise and smoking cessation also markedly lower lipid levels and help prevent lipodystrophy [60]. It is also recommended that the patient's glucose and lipid concentrations be monitored regularly [59,60]. Current guidelines should be followed for patients with dyslipidemia for primary and secondary risk prevention. Known drug interactions should be avoided, such as that with simvastatin and ritonavir, which can lead to a 400-fold increase in simvastatin concentrations [61,62].

### Left ventricular systolic dysfunction

#### Incidence

Before HAART therapy, the two-to-five-year incidence of symptomatic heart failure ranged from 4 to 28% in HIV patients, suggesting a prevalence of symptomatic HIV-related heart failure of between 4 and 5 million cases worldwide [5,6]. The incidence of clinically important cardiac disease in HIV-infected patients has been markedly reduced by HAART. However, HAART is only available to a minority of those in need [6,63–65].

Patients receiving HAART also frequently have asymptomatic abnormalities in cardiac structure [66]. Echocardiographic measurements indicate 18% have LV systolic dysfunction, 6.5% have LV hypertrophy, and 40% have left atrial dilation [64]. A history of MI, current tobacco smoking, and elevated highly sensitive C-reactive protein were associated with LV systolic dysfunction [64].

During pre-HAART era HIV-infected children aged ten or younger, 25% died from chronic cardiac disease [5,6], and 28% experienced serious cardiac events after an AIDS-defining illness [1,5,6,65]. Increased mortality is only associated with a mild decrease in LV systolic function or an increase in LV mass in children [24]. The NHLBI-funded Highly Active Antiretroviral Therapy-Associated Cardiotoxicity (CHAART-II) study collected longitudinal echocardiographic measurements in HIV-infected children and adolescents exposed to HAART or multi-drug ART. When compared to HIV-infected but relatively less ART-exposed children from the HIV-infected cohort from the Pediatric Pulmonary and Cardiovascular Complications of Vertically Transmitted HIV Infection (P^2^C^2^ HIV) study, the CHAART-II patients had persistently decreased LV mass. Although in infancy, the CHAART-II patients had significantly better LV contractility compared to the P^2^C^2^ HIV group, at ten years of follow-up, LV contractility significantly decreased in the CHAART-II group to a level equivalent to decreased LV systolic function in infancy in the P^2^C^2^ HIV group. These findings suggest that long-term HAART exposure may be cardioprotective for a finite period early in life, but decreases as this HIV-infected population ages into adolescence and early adulthood. Further longitudinal follow-up studies are needed in adolescents and young adults who were perinatally infected with HIV to better characterize their future cardiac risk. After 11 years of HAART exposure, in CHAART-II patients, LV function was equivalent to that of the HAART-unexposed P^2^C^2^ HIV-infected cohort. The conclusion was that the protective effects of HAART exposure on cardiac function appeared to diminish 11 years after exposure. A larger, but otherwise similar HIV-infected paediatric cohort from the NIH-funded Pediatric HIV/AIDS Cohort Study's Adolescent Master protocol required only a single echocardiogram. Generally, measures of LV structure and function were better in this long-term HAART-exposed group than in the relatively HAART-unexposed P^2^C^2^ HIV cohort, but were not as normal as those in an HIVEU control group [67]. Although the general conclusion was that HAART exposure in HIV-infected children appeared to be cardioprotective, the cross-sectional study could not support conclusions regarding the long-term trajectories of cardiac health or dysfunction. Serial echocardiographic and other cardiovascular risk screening in this cohort as they age could inform the long-term cardiovascular risk in perinatally HIV-infected children in the HAART era.

The CHAART-I study collected serial echocardiograms in a cohort of HIVEU children exposed perinatally to either multi-drug ART or HAART [68]. At age two, these children had below-normal LV mass, LV dimension, and septal wall thickness, indicating smaller hearts. In contrast, LV function was increased. These differences were more pronounced in girls [68]. In a larger cohort of HIVEU, perinatally HAART-exposed and slightly older (aged 3–5 years) children from the PHACS SMARTT protocol, preliminary results from a single echocardiogram showed that 16% of children had at least one abnormal echocardiographic measure. First trimester exposure to various ART agents was associated with specific echocardiographic abnormalities. For instance, first trimester exposure to abacavir was associated with decreased LV wall thickness. In a separate study of the PHACS SMARTT HIVEU cohort, serum cardiac biomarker measurements suggested that HIVEU children perinatally exposed to multiple ART agents might have subclinical myocardial inflammation. Specifically, abacavir exposure was potentially associated with deleterious cardiac effects [69].

The results of cardiac biomarkers in the PHACS AMP HIV-infected cohort are still being analyzed and could provide further insights into both the long-term pathophysiologic effects of HAART exposure as well as how best to evaluate long-term cardiovascular risk. Currently, additional analyses are on-going comparing the cross-sectional echocardiographic measures in this PHACS SMARTT cohort to the relatively ART-unexposed P^2^C^2^ HIV cohort and the smaller but longitudinally followed CHAART-I perinatally HAART-exposed cohort. The results of these on-going analyses may better elucidate the effects of prenatal HIV and ART exposures on cardiac measures of structure and function in HIVEU children.

Recent data show a marked decline in the incidence of both clinical cardiomyopathy and structural abnormalities and an apparent cardioprotective effect of HAART in children and adolescents [58,63–65,68].

#### Clinical presentation

Concurrent pulmonary infections, anaemia, pulmonary hypertension, malnutrition, portal hypertension, and malignancy can modify or confuse the distinctive signs in HIV-infected patients that define heart failure in other populations. Patients can present with LV systolic dysfunction that is anywhere from asymptomatic to New York Heart Association Class III (marked functional limitations) or IV heart failure (severe functional limitations) [62].

Echocardiography, including strain measurements, and cardiac magnetic resonance imaging is useful for assessing LV function, in addition to diagnosing LV dysfunction. Images often reveal LV hypertrophy, dilation, or low-to-normal wall thickness, as well as left atrial dilation [5,24,33,62,64]. Echocardiographic assessment is recommended at baseline and every 1–2 years thereafter, or as indicated, in any patient at elevated cardiovascular risk who has unexplained or persistent pulmonary symptoms or viral co-infections or with any clinical manifestations of CVD [5,54,62,64].

Electrocardiography (ECG) often reveals nonspecific conduction defects or repolarization changes in ART naïve patients. Chest radiography has low sensitivity and specificity for diagnosing heart failure in HIV-infected patients treated or untreated with ART. Several small studies of HIV-infected individuals revealed that blood brain natriuretic peptide concentrations were inversely correlated with LV ejection fraction. This inverse correlation can be useful in the differential diagnosis of congestive cardiomyopathy in HIV-infected patients [33,69,70].

Progressive LV dilation is common in children infected with HIV. LV dilation may precede heart failure (five-year cumulative incidence, 12.3%) and is associated with elevated LV afterload, LV hypertrophy, and reduced LV function [65]. Early and continuous treatment with HAART for at least five years in HIV-infected children prevented clinically important heart failure better than in earlier groups and preserved cardiac structure and function, indicating that HAART may be cardioprotective [65].

Both the PHACS and CHAART studies suggest that any cardiac changes in the HAART era are generally subclinical in children. Further, in addition to characterizing lifetime ART exposure, traditional non-HIV cardiovascular risk factors, will be needed to best determine differences in global cardiovascular risk between perinatally HIV-infected and HIVEU children, and that in the general population.

#### Pathogenesis in children

Two mechanisms of pathogenesis have been described in children treated with ART in the pre-HAART with perinatally-transmitted HIV infection: dilation of the LV with a reduced ratio of the LV wall thickness to end-systolic dimension and concentric hypertrophy of the muscle and dilation, in which the ratio of LV thickness to end-systolic dimension remains normal or is increased [5].

#### Pathogenesis in young adults

As these children enter young adulthood, adult pathogenesis of the disease becomes more relevant. Several causative agents have been postulated for HIV-related cardiomyopathy in children and adults receiving treatment who are from the pre-HAART era (Table 1) [62,65,68].

#### Myocarditis

Dilated cardiomyopathy can be related to the direct action of HIV on myocardial tissue or to proteolytic enzymes or cytokine mediators induced by HIV alone or with co-infecting viruses [71]. Endomyocardial biopsy specimens have revealed *Toxoplasma gondii*, coxsackievirus group B, Epstein-Barr virus, cytomegalovirus, adenovirus, and HIV in myocytes. Further research is required to determine if these co-infecting agents also apply in the post-HAART era.

Only scant and patchy inflammatory cell infiltrates in the myocardium have been identified in autopsy and biopsy findings [5,62,71,72], indicating that HIV can infect myocardial interstitial cells and are rarely found in cardiomyocytes. Patients with confirmed myocarditis have an increased number of infected interstitial cells where proteolytic enzymes or increased concentrations of TNF-α or interleukin may injure the myocytes. Studies have revealed that these affected patients have increased concentrations of TNF-α, inducible nitric oxide synthase, and IL-6 [5,62,71,73]. About 40% of patients with HIV-related cardiomyopathy have no opportunistic infection before the onset of cardiac symptoms [5,6], although this cardiomyopathy is commonly not associated with specific opportunistic infections.

#### Cytokine alterations

Increased TNF-α production induced by HIV infection can elevate nitric oxide production and alter intracellular calcium homeostasis, transforming growth factor-β and endothelin-1 activity [74]. When nitric oxide concentrations were elevated experimentally, myocytes were killed or injured, causing negative inotropic effects [74]. Clinical trials are needed in order to determine the effect of cytokine alterations in the current post-HAART era.

#### Nutritional deficiencies

Nutritional deficiencies are common in HIV-infected individuals, particularly in the late stages and in young infants. Electrolyte imbalances and deficiencies in elemental nutrients are often a result of diarrhoea and poor absorption. Deficiencies of trace elements have been associated with cardiomyopathy. For example, coxsackievirus is more virulent in selenium-deficient cardiac tissue [54]. Left ventricular function is restored and cardiomyopathy is reversed with selenium replacement. Concentrations of vitamin B12, carnitine, growth hormone, and thyroid hormone can be altered in HIV disease; all have been associated with LV dysfunction [69,75].

#### Course of disease

Patients with asymptomatic LV dysfunction, defined as a LV fractional shortening <28% with global LV hypokinesis, may have echocardiographically defined transient disease. One serial echocardiographic study reported that three out of six patients with abnormal LV fractional shortening had normal readings after a mean of nine months. The three patients with persistently depressed LV function all died within one year of diagnosis of LV systolic dysfunction [5].

#### Prognosis

Mortality has increased in HIV-infected patients with cardiomyopathy, independently of CD4 count, sex, age, or HIV risk group. In the pre-HAART era, median survival from diagnosis to AIDS-related death was 101 days in patients with LV dysfunction. Patients with normal hearts had a median survival of 472 days at a similar stage of infection [1,5]. Neither isolated right ventricular dysfunction nor borderline LV dysfunction increased the risk of AIDS-related death.

**Figure 2:**
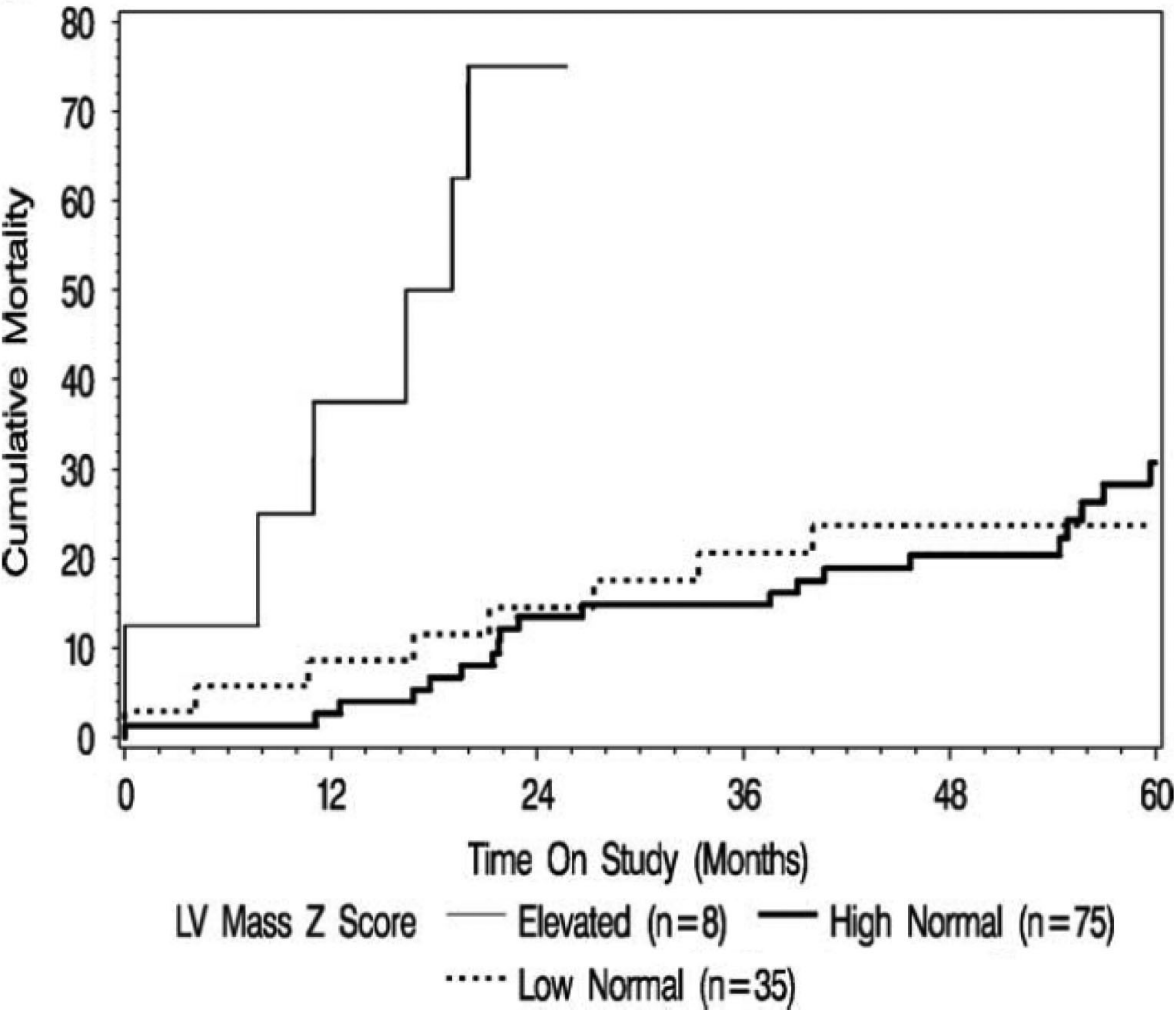
Mildly increased LV mass is a risk marker for early HIV mortality even though it is still inadequate for LV dimension. Reproduced with permission from ref. 24.

In the P^2^C^2^ HIV study, the median age was 2.1 years and five-year cumulative survival was 64% [5]. Children with baseline measurements showing depressed LV fractional shortening or increased LV dimension, mass, thickness, heart rate, blood pressure, or wall stress had a higher mortality. Increased LV wall thickness and decreased LV fractional shortening also predicted adjusted survival (Figure 2) [5]. Although increased LV wall thickness identified a population at risk only 18–24 months before death, LV fractional shortening was abnormal for three years before death. Although most patients received zidovudine at some point during the P^2^C^2^ study, a separate report found that zidovudine was not associated with cardiac complications [76]. Thus, LV fractional shortening may be a useful long-term predictor of mortality, and LV wall thickness, a useful short-term predictor in children receiving ART from the pre-HAART era [5,24,65,77].

In the P^2^C^2^ HIV-infected cohort, echocardiographic evidence of increased LV mass was associated with post-mortem cardiomegaly and documented chronically increased heart rate before death but not with anaemia, HIV viral load, or encephalopathy [5]. Mild persistent depression of LV function and elevated LV mass were associated with higher all-cause mortality in children infected with HIV [24,65,68]. A reduction in LV fractional shortening from 34 to 30% in a ten-year old, equivalent to a reduction of 2 Z-scores, is associated with an increase from 15 to 55% in five-year mortality [65,68]. Fractional shortening was higher in HIV-uninfected children of HIV-infected mothers with *in utero* exposure to ART than in HIV-uninfected children of HIV-infected mothers unexposed to ART. However, exposure to ART was associated with decreased LV mass, LV dimension, and septal thickness [68]. Any exposure to HAART in perinatally-infected children with HIV markedly affects LV mass, LV contractility, and LV afterload [78]. Rapid-onset heart failure has a grim prognosis in both HIV-infected children and adults. More than half of patients die from primary cardiac failure within a year of presentation [1,5,62].

#### Therapy

Similar to non-ischemic cardiomyopathy, therapy for dilated cardiomyopathy associated with HIV infection includes diuretics, digoxin, aldosterone antagonists, β-blockers, and angiotensin-converting enzyme inhibitors, as tolerated. The efficacy of specific cardiac therapeutic regimens other than intravenous immunoglobulin is unknown [2]. Due to low systemic vascular resistance, patients may be very sensitive to angiotensin-converting enzyme inhibitors. Preventing heart failure using HAART remains the best treatment [59,60,62].

Infections should be treated to improve or resolve related cardiomyopathy. Right ventricular biopsy may assist in target therapy in addition to identifying infectious causes of failure [62]. Right ventricular biopsy may be underused [6,62,64,71].

**Figure 3:**
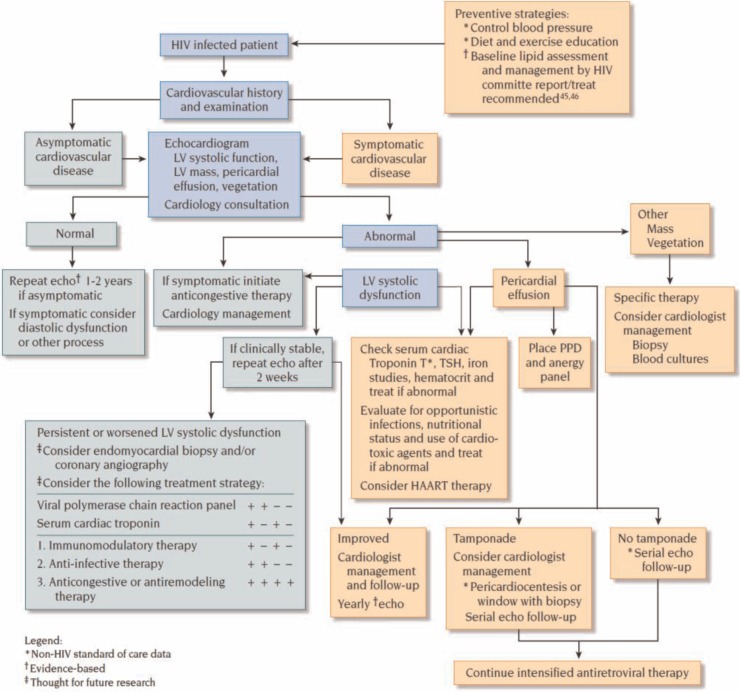
Cardiac dysfunction in HIV-infected patients. HAART=highly active antiretroviral therapy; LV=left ventricular; PPD=purified protein derivative; TSH=thyroid-stimulating hormone. Reproduced with permission from “Fisher SD, Lipshultz SE. Chapter 72: Cardiovascular abnormalities in HIV-infected individuals. In: Braunwald's Heart Disease: A Textbook of Cardiovascular Medicine, Ninth Edition. Editors: Bonow RO, Mann DL, Zipes DP, Libby P. Philadelphia: Elsevier Saunders. 1618–27. 2011 ISBN: 978-1-4377-0398-6.”

Serial echocardiographic measurements should be performed at clinically relevant intervals, such as four months, after medical therapy is begun. Monitoring recommendations for testing and timing of follow-up are based on studies relating impaired LV fractional shortening to a worse prognosis. A biopsy should be considered if cardiac function continues to deteriorate or if the clinical course worsens. Patients with heart failure who have not responded to two weeks of medical therapy may benefit from cardiac catheterization and endomyocardial biopsy, which may reveal lymphocytic infiltrates suggesting myocarditis or treatable opportunistic infections (by special stains), permitting aggressive therapy of an underlying pathogen [5,52,62,68,71,74]. Angiography should be performed selectively if there are risk factors for atherosclerotic disease or suggestive clinical symptoms (Figure 3) [33,44].

In HIV-uninfected children, intravenous immunoglobulins help treat acute congestive cardiomyopathy and nonspecific myocarditis. Monthly immunoglobulin infusions have minimized LV dysfunction, increased LV wall thickness, and reduced peak LV wall stress in HIV-infected children, suggesting that both impaired myocardial growth and LV dysfunction can be immunologically mediated [2].

Although transplantation therapy is not widely available, it remains an area of active research and has been successfully performed [70].

#### Animal models

Exposure to a ubiquitous environmental agent, heat-killed *Mycobacterium avium* complex, results in exaggerated myocardial pathology in Rhesus macaques infected with simian immunodeficiency virus. In this model, enternacept (a TNF antagonist) prevented LV dysfunction, suggesting a TNF-α-dependent pathway in the development of cardiomyopathy in HIV infection [74].

### Left ventricular diastolic dysfunction

Diastolic dysfunction is relatively common in long-term survivors of HIV infection, as suggested by clinical and echocardiographic data. Such LV dysfunction may precede LV systolic dysfunction and mark an early manifestation of HIV-associated cardiac disease [18,75,79–81]. However, LV diastolic function has not been characterized in HIV-uninfected children exposed *in utero* to ART. Slower LV relaxation during diastole leads to a decrease in early diastolic filling. Left ventricular compliance decreases as LV diastolic dysfunction worsens and left atrial pressure increases. Moderate-to-severe LV diastolic dysfunction, an independently predicts mortality, regardless of normal LV systolic function [82]. The clinical impact of LV diastolic function has been studied in children with cardiomyopathy along with other co-morbidities, such as obesity, generalized autoimmune disease, and diabetes [83–88].

In one cross-sectional study, early diastolic mitral valve annular velocity was lower in HIVEU children born to HIV-infected mothers who were exposed *in utero* to ART compared to a group of HIV-uninfected children born to HIV-uninfected mothers with no perinatal ART exposure. In addition, lower early diastolic mitral valve annular velocity was associated with lower maternal CD4 counts in the final trimester [89]. The longitudinal CHAART-I study found subclinical LV diastolic abnormalities in both LV compliance and relaxation among HIVEU children exposed perinatally to multi-drug ART [83]. In a study of 656 asymptomatic HIV-infected adults, 26% had screening echocardiographic evidence of LV diastolic dysfunction [64]. Adults with LV diastolic dysfunction, compared to those without, were older, tended to have higher body mass indexes, more likely to have hypertension, and had been infected longer [81]. Whether LV diastolic dysfunction is associated with an increased risk of early coronary disease is unknown [75,81]. In children, also unknown is the clinical importance of LV systolic versus diastolic dysfunction in HIV-infected and HIVEU children perinatally exposed to multi-drug ART. The temporal occurrence of these LV systolic versus diastolic echocardiographic changes is important in determining the effects of HIV exposure and ART exposure.

Uncontrolled HIV replication and ART increase IL-6 concentrations [90]. Viral proteins or replication in the myocardial macrophages in animal models may cause LV diastolic dysfunction. Longitudinal mitral inflow and tissue Doppler echocardiographic studies of Rhesus macaques infected with simian immunodeficiency virus found that LV diastolic dysfunction was common and strongly correlated with the extent of viral replication in the myocardium [90].

### Pulmonary hypertension

Pulmonary arterial hypertension (PAH) occurs in about 0.5% of HIV-infected patients. This does not include cases of elevated pulmonary pressure secondary to interstitial lung disease or chronic obstructive pulmonary disease where the pathophysiology and response to therapies differ. The introduction of HAART has not changed the prevalence of pulmonary arterial hypertension [3,91–94]. In HIV-infected patients, normal endothelial structure is replaced by plexogenic pulmonary arteriopathy, which is characterized by remodelling of the pulmonary vasculature with intimal fibrosis [92,93]. Perfusion scans are normal and lung fields may be clear on examination and chest radiographs [92]. Pulmonary arterial hypertension has been reported in HIV-infected patients without a history of thromboembolic disease, intravenous drug use, or pulmonary infections associated with HIV [3,93,94].

Primary pulmonary hypertension has been found in patients with haemophilia receiving lyophilized factor VIII, intravenous drug users, and patients with LV dysfunction, obscuring any relationship with HIV [3,93]. Whether PAH is associated with human herpesvirus 8 is unclear. HIV or a co-infection might cause endothelial damage and mediator-related vasoconstriction of the pulmonary arteries.

Two recent studies found that CD4 count was independently associated with survival in 154 patients with HIV and PAH, with pulmonary hypertension as the direct cause of death in 72% of those affected. Survival rates at one, two, and three years were 73, 60, and 47%, respectively. Survival rates in New York Heart Association functional Class III–IV patients at the time of diagnosis were 60, 45, and 28% at one, two, and three years [93,94], respectively. In one year, 52% of 549 patients with HIV and PAH died with 51% from right heart failure [94].

Standard treatments for PAH, such as PDE-5 inhibitors, endothelin antagonists, and prostacyclin analogues, have been effective in HIV-infected patients. Therapy also includes anticoagulation (on the basis of individual risk-benefit analysis) [92]. Affected patients have continued HAART. In patients with HIV and PAH, PAH should be aggressively treated because it is life-threatening as set forward in the American College of Cardiology Foundation's treatment guidelines for PAH [92]. Morbidity and mortality seem to be caused by PAH more than by HIV infection and, therefore, should be clinically managed based on current recommendations from the American College of Cardiology expert consensus document on pulmonary hypertension [92].

### Pericardial effusion

#### Incidence

Pericardial effusions were found in up to 11% of patients with AIDS before the HAART era. The prevalence of effusion in asymptomatic AIDS patients reaches a mean of about 22% after 25 months, rising over time [95]. In a recent study, only 2 out of 802 HAART-treated patients had clinically important effusions, indicating the greatly reduced incidence with treatment of HIV [95].

#### Clinical presentation

HIV-infected patients with pericardial effusions generally have lower CD4 counts than those without effusions [92]. Effusions are generally small and asymptomatic. HIV infection should be suspected whenever a patient presents with unexplained pericardial effusion or tamponade. In a retrospective series from a city hospital, 13 out of 37 (35%) patients with cardiac tamponade had HIV infection [95]. Although rare, tuberculosis has been found as a presenting infection for pericardial effusions in underdeveloped areas where tuberculosis is prevalent [95,96]. These cases have therapeutic implications and deserve special attention [97].

#### Pathogenesis

Pericardial effusion is often part of a generalized serous effusive process also involving pleural and peritoneal surfaces. Enhanced cytokine production in AIDS may be associated with this “capillary leak”. Other well-described associations (Table 1) include uremia from HIV-associated nephropathy or drug nephrotoxicity. Effusion nearly triples the risk of death among AIDS patients [95]. Immune reconstruction inflammatory syndrome can cause pericardial effusions and pericarditis in patients co-infected with HIV and tuberculosis [98]. Pericardiocentesis has been found to be a safe and effective treatment of tuberculosis pericardial effusions in HIV-infected patients [99].

#### Monitoring and therapy

Baseline echocardiography and ECG measurements should be taken on all HIV-infected patients with evidence of heart failure, Kaposi's sarcoma, tuberculosis, or other pulmonary infections. Pericardiocentesis is indicated for pericardial effusion when there are clinical signs of tamponade (such as elevated jugular venous pressure, dyspnea, hypotension, persistent tachycardia, or pulsus paradoxus), or echocardiographic signs of tamponade (such as continuous-wave Doppler echocardiographic evidence of respiratory variation in valvular inflow, septal bounce, right ventricular diastolic collapse, and a large effusion).

Patients with pericardial effusion without tamponade should be evaluated for malignancy and opportunistic infections, such as tuberculosis. HAART should be considered if it has not already been instituted. Repeat echocardiography is recommended after one month or sooner if indicated (Figure 3) [20,62].

## Infective endocarditis

Infective endocarditis has been reported in adults with HIV infection, most commonly in intravenous drug users, and usually causes right-sided endocarditis. The most common organism associated with endocarditis in HIV-infected adult patients is Staphylococcus aureus. Endocarditis caused by *Aspergillus fumigatus*, *Candida* species, and *Cryptococcus neoformans* are more common in intravenous drug users with HIV than in those without HIV. Generally, HIV does not appear to significantly influence the response to treatment or outcome (Table 1) [20].

Late-stage AIDS patients with poor nutritional status and severely compromised immune systems may experience a more fulminant course and a higher mortality. However, several patients have been successfully treated with antibiotics. Surgical indications in HIV-infected patients with endocarditis include persistent bacteremia despite intravenous antibiotics to which the organism is sensitive, hemodynamic instability, persistent embolization and severe valvular destruction in patients with a reasonable life expectancy after surgery.

Endocarditis in HIV-infected children is rare. There is a report of a two month old HIV-infected Ugandan boy who presented with disseminated Staphylococcus aureus infection with a large obstructing vegetation on the free wall of the left ventricle in association with a purulent pericardial effusion and an empyema. Echocardiogram showed no structural abnormalities other than a patent foramen ovale [100].

## Nonbacterial thrombotic endocarditis

Marantic or non-bacterial thrombotic endocarditis involves deposition of large, friable, sterile vegetations predominantly on the cardiac valves. These vegetations have been associated with disseminated intravascular coagulation and systemic embolization. Vegetations are rarely diagnosed before death, but when they are, clinically important emboli are likely [20]. Marantic endocarditis is rare in children, but was described in a child newly diagnosed with HIV at age 14 months. The child developed pneumonia, Staphylococcus sepsis, and later developed acute cardiac failure with valvular dysfunction, hepatosplenomegaly, ascites and failure to thrive. An echocardiogram showed bright echoes within the chordea of the tricuspid valve and the tips of the leaflets. After a complicated course, the child died of pulmonary insufficiency at age 34 months [101]. It is likely that this type of endocarditis is more likely to be identified in patients with delayed HIV diagnosis, limited or no access to ART and those with progressive disease. In the early HIV epidemic, several case series in adults suggested a high incidence of this uncommon disorder; however, few cases have since been reported.

## Cardiovascular malignancy

Malignancy affects many adult AIDS patients, generally in the later stages of disease. Cardiac malignancy may be a primary tumour or a metastatic secondary site. Although lymphomas have been associated with malignancy in HIV-infected children, the incidence is low and cardiac malignancy is rare in children with HIV infection. The Children's Cancer Group and the Paediatric HIV Clinic at the National Cancer Institute reported 65 tumours diagnosed between 1982 and 1997 in 64 HIV-infected children [102], although these patients were not on treatment. Non-Hodgkin's lymphoma accounted for 65% of these tumours. In this study, almost one-third of the children with this disease had normal or moderate immune suppression. Leiomyosarcoma occurred in 17% and Kaposi's sarcoma in 5%.

Kaposi's sarcoma (angiosarcoma) affected up to 35% of AIDS patients early in the HIV epidemic and is associated with human herpesvirus 8. Its incidence is inversely related to CD4 count. Although sarcoma is infrequently described as a primary cardiac tumour, autopsy studies have found that 28% of HIV-infected patients with widespread Kaposi's sarcoma had cardiac involvement [4]. Kaposi's sarcoma is often an endothelial cell neoplasm with a predilection in the heart for sub-pericardial fat around the coronary arteries [4,20]. Combination antiretroviral therapy has markedly decreased the incidence of Kaposi's sarcoma from that in the pre-HAART era [20].

Children with HIV infection may harbour human herpesvirus 8, the virus associated with Kaposi's sarcoma. Kaposi's sarcoma is endemic in eastern equatorial Africa. It can cause a lymphadenopathic type of Kaposi's sarcoma that is found mainly in children, which may have a fulminant course and ultimately also invade organ systems. Two children were reported in the United States early in the epidemic, both died before one year of age and had progressive HIV infection with severe immune deficiency. There were lesions of Kaposi's sarcoma in the lymph glands and spleen and in one case in the thymus [103].

Primary cardiac malignancy associated with HIV infection is generally caused by cardiac lymphoma. Lymphoma, an AIDS-defining illness, has a higher incidence in HIV-infected populations. Non-Hodgkin's lymphomas are 25–60 times more common in HIV-infected individuals. They are the first manifestation of AIDS in up to 4% of new cases [4]. This disease is not specifically associated with severe immune suppression. Patients with primary cardiac lymphoma can present with signs of heart failure, chest pain, or arrhythmias. Cardiac lymphoma can cause rapid progression to cardiac tamponade, heart failure, myocardial infarction, tachyarrhythmias, conduction abnormalities, or superior vena cava syndrome. Malignant cells can be found in the pericardial fluid. Systemic multi-agent chemotherapy with and without concomitant radiation or surgery has benefitted some patients, but overall, the prognosis is poor [4]. Treatment with HAART has not substantially affected the incidence of HIV-related non-Hodgkin's lymphomas [20,104].

### Isolated right ventricular disease

Isolated right ventricular hypertrophy is rare in HIV-infected individuals, with or without right ventricular dilation. It is generally related to pulmonary disease that increases pulmonary vascular resistance. Possible causes include pulmonary arteritis from the immunological effects of HIV disease, multiple bronchopulmonary infections, or microvascular pulmonary emboli caused by thrombus or contaminants in injected drugs such as Talc [3]. Right ventricular diastolic dysfunction has been reported in asymptomatic patients studied with Doppler tissue imaging [105].

## Vasculitis

Vasculitis may occur in patients with fever of unknown origin, unexplained arthritis or myositis, unexplained multisystem disease, glomerulonephritis, or peripheral neuropathy (especially mononeuritis multiplex), and in unexplained gastrointestinal, cardiac or central nervous system ischemia. Several types have been described in HIV-infected patients, but all types show diffuse inflammation of the vessel walls [106]. Successful immunomodulatory therapy has been reported, chiefly with systemic corticosteroid therapy [106]. The HIV protein, transactivator of transcription (Tat), has been implicated in the pathogenesis of vasculitis [106].

## Sudden cardiac death

Sudden cardiac death is becoming increasingly common as the HIV-infected population ages. In one study, sudden cardiac death accounted for 86% of all cardiac-related deaths (30 of 35). The mean rate of sudden cardiac death was 2.6 per 1000 person-years (95% confidence interval: 1.8–3.8), which was 4.5-fold as high as that expected in an age-matched uninfected population [107]. One report found that patients dying from sudden cardiac death were older than those dying from AIDS (mean age at death, 49 vs. 45 years, *p*=0.02), had a higher prevalence of prior MI (17% vs. 1%, *p*<0.001), cardiomyopathy (23% vs. 3%, *p*<0.001), heart failure (30% vs. 9%, *p*=0.004), and arrhythmias (20% vs. 3%, *p*=0.003) [107].

## QT interval and PR prolongation

HIV infection is associated with QT prolongation and Torsades de Pointes ventricular tachycardia. There is an increased risk of sudden death late in HIV infection and specifically with AIDS. The incidence of QT prolongation increases as the disease progresses to AIDS [108]. Hepatitis C is independently associated with increased QT duration. One study found that the risk of QT prolongation (that is, QTc values of 470 ms or higher) was 16% with HIV alone and 30% with both HIV and hepatitis C infections [109]. The risk of increased QT duration is also higher in patients treated with ART as well as anti-tuberculosis medications, such as levofloxacin, moxifloxacin, and bedaquoline [51].

Different protease inhibitor-based regimens have a similar, minimal effect on the QT interval, but significantly prolong the PR interval by a difference of 3 ms in non-boosted protease inhibitor regimen to 5.11 ms in boosted protease inhibitor regimen. The intervals do normalize on withdrawal of the protease inhibitor therapy and prolongation is not associated with NNRTIs. The clinical significance is not well established [110]. It is thought that PR prolongation may lead to a higher likelihood of complete heart block during immune reconstitution inflammatory syndrome during initiation of ART.

## Autonomic dysfunction

**Figure 4:**
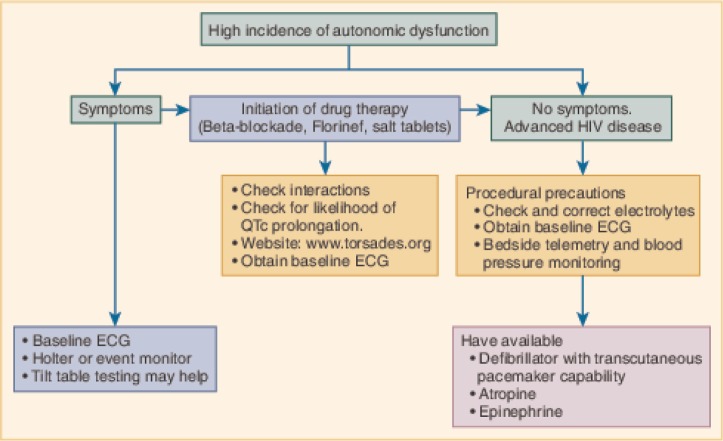
Evaluation and management of dysautonomia. ECG = electrocardiography. Reproduced with permission from “Fisher SD, Lipshultz SE. Chapter 72: Cardiovascular abnormalities in HIV-infected individuals. In: Braunwald's Heart Disease: A Textbook of Cardiovascular Medicine, Ninth Edition. Editors: Bonow RO, Mann DL, Zipes DP, Libby P. Philadelphia: Elsevier Saunders. 1618–27. 2011 ISBN: 978-1-4377-0398-6.”

Preliminary clinical signs of autonomic dysfunction in HIV-infected patients include syncope and presyncope, diarrhoea, diminished sweating, bladder dysfunction, and impotence. One study found heart rate variability Valsalva ratio, cold pressor testing, hemodynamic responses to isometric exercise, tilt-table testing, and standing showed that autonomic dysfunction occurred in HIV-infected individuals and was pronounced in AIDS patients. AIDS patients receiving HAART were relatively protected. Patients with HIV-associated nervous system disease had the greatest abnormalities in autonomic function (Figure 4) [111].

## Complications of therapy

Antiretroviral medications have greatly reduced mortality by delaying the progression to AIDS and increased quality of life of HIV-infected patients [52]. However, these same therapies are associated with a number of complications [20,57,80,112,113].

As previously detailed, altered body composition and hyperlipidemia are associated with PIs. Nucleoside reverse transcriptase inhibitors may lead to increasing the child's cardiometabolic risk [112,113]. Lipid abnormalities vary with different PIs. Ritonavir had the most adverse effects on lipids, with a mean increase in total cholesterol concentration of 2.0 mmol/L and a mean increase in triglyceride concentration of 1.83 mmol/L [57,80,114]. More modest increases of total cholesterol concentration without marked triglyceride increases were found in patients taking indinavir and nelfinavir. Combination with saquinavir (including atanazavir and saquinavir in salvage therapy) did not further elevate total cholesterol concentrations. Protease inhibitors significantly increased lipoprotein (a) in patients with elevated pre-treatment values (>20 mg/dL), which is another risk marker for atherosclerotic cardiovascular disease [57,80,114]. In some cases, switching PIs may reverse both elevations in triglyceride concentrations and abnormal fat deposition. Low-level aerobic exercise may also help reverse lipid abnormalities [20,53]. Zidovudine or azidothymidine (AZT) has been implicated in skeletal muscle myopathies. In culture, AZT causes a dose-dependent destruction of human myotubes. Human cultured cardiac muscle cells treated with AZT developed mitochondrial abnormalities, and nucleoside reverse transcriptase inhibitors in general have been associated with altered mitochondrial DNA replication and cardiac structure [20,115]; it is uncertain whether altered mitochondrial DNA replication is the cause of cardiomyopathy. However, cardiac myopathies have not been evident in clinical data. Some patients with LV dysfunction may improve when AZT therapy is stopped [20]. Some evidence has been presented associating ARTs and mitochondrial toxicity [116]. This and additional factors may predispose children infected with HIV to reduced aerobic capacity. HIV-infected children and adolescents had lower cardiorespiratory fitness, lower extremity strength, and flexibility than did their uninfected counterparts. Additionally, HAART exposure for greater than five-years and higher total body fat percentage independently had a negative effect on aerobic capacity [60].

Intravenous pentamidine, used to treat *Pneumocystis jirovecii* pneumonia in patients intolerant of trimethoprim-sulfamethoxazole, has been associated with Torsades de Pointes and refractory ventricular tachycardia [20]. Pentamidine should be reserved for patients with a QTc interval below 480 ms. Multiple medication reactions and interactions have occurred during HIV treatment and are a major cause of cardiac emergencies in HIV-infected patients (Table 3) [62,65,117].

**Table 3:** Cardiac interactions and side effects of drugs commonly used in HIV therapy

Class	Cardiac drug interactions	Cardiac side effects
*Antiretroviral* Nucleoside (and nucleotide) reverse transcriptase inhibitors Abacavir (ABC), Didanosine (ddI), Emtricitabine (FTC) Lamivudine (3TC), Stavudine (d4T), Tenofovir (TDF), Zalcitabine (ddC), Zidovudine (ZDV, AZT)	Zidovudine and dipyridamole Stavdine and DDI	Rare: lactic acidosis, hypotension Accelerated risk with cardiopulmonary bypass Zidovudine: skeletal muscle myopathy, myocarditis Mitochondrial toxicity with lipodystrophy
Non-nucleoside reverse transcriptase inhibitors Delavirdine (DLV), Efavirenz (EFV), Nevirapine (NVP), Rilpivirine (RPV)	Calcium channel blockers, warfarin, β-blockers, nifedipine, quinidine, steroids, theophylline. Delavirdine can cause serious toxic effects if given with antiarrhythmic drugs and calcium channel blockers	Arrhythmia
Protease inhibitors Amprenavir (APV), Atazanavir (ATV), Darunavir (DRV), Fosamprenavir (FPV) Indinavir (IDV), Lopinavir/ritonavir (LPV/r), Nelfinavir (NFV), Ritonavir (RTV), Saquinavir (SQV), Tipranavir (TPV)	Metabolized by cytochrome P450 and interact with other drugs metabolized through this pathway, such as selected antimicrobials, antidepressant and antihistamine agents, cisapride, HMG CoA reductase inhibitors (lovastatin, simvastatin), and sildenafil. Potentially dangerous interactions that require close monitoring or dose adjustment can occur with amiodarone, disopyramide, flecainide, lidocaine, mexiletine, propafenone, and quinidine. Ranolazine (1.8–2.3× increase in Ranolazine level) Ritonavir is the most potent cytochrome activator (CYP3A) and P-glycoprotein inhibitor and is most likely to interact. Indinavir, amprenavir, and nelfinavir are moderate. Saquinavir has the lowest probability to interact Calcium channel blockers, prednisone, quinine, β-blockers (1.5- to 3-fold increase). Decreases theophylline concentrations	Implicated in premature atherosclerosis, dyslipidemia, insulin resistance, diabetes mellitus, fat wasting, and redistribution Abacavir may be associated with increased risk of MI^13^
Integrase strand transfer inhibitors (INSTIs) Elvitegravir (EVG), Raltegravir (RAL)		
CCR5 antagonists Maraviroc		
Fusion inhibitor Enfuvirtide	–	
Anti-infective antibiotics	*Rifampin:* Reduces therapeutic effect of digoxin by inducing intestinal P-glycoprotein, reduces protease inhibitor concentration and effect *Erythromycin:* Cytochrome P450 metabolism and drug interactions *Trimethoprim-sulfamethoxazole:* (Bactrim) increases warfarin effects	*Erythromycin:* Orthostatic hypotension, ventricular tachycardia, bradycardia, Torsades (with drug interactions) *Clarithromycin:* QT prolongation and Torsades de Pointes *Trimethoprim-sulfamethoxazole:* Orthostatic hypotension, anaphylaxis, QT prolongation, Torsades de Pointes, hypokalemia *Sparfloxacin (fluoroquinolones):* QT prolongation
Antifungal agents	*Amphotericin B:* Digoxin toxicity *Ketoconazole or itraconazole:* Cytochrome P450 metabolism and drug interactions—increases levels of sildenafil, warfarin, HMG CoA reductase inhibitors, nifedipine, digoxin	*Amphotericin B:* Hypertension, arrhythmia, renal failure, hypokalemia, thrombophlebitis, bradycardia, angioedema, dilated cardiomyopathy. Liposomal formulations still have the potential for electrolyte imbalance and QT prolongation *Ketoconazole, fluconazole, itraconazole:* QT prolongation and torsades de pointes
Antiviral agents	*Ganciclovir:* Zidovudine	*Foscarnet:* Reversible cardiac failure, electrolyte abnormalities *Ganciclovir:* Ventricular tachycardia, hypotension
Antiparasitic		*Pentamidine:* Hypotension, QT prolongation, arrhythmias (Torsades de Pointes), ventricular tachycardia, hyperglycemia, hypoglycemia, sudden death. These effects are enhanced by hypomagnesemia and hypokalemia
Chemotherapy agents	*Vincristine, doxorubicin:* Decrease digoxin level	*Vincristine:* Arrhythmia, myocardial infarction, cardiomyopathy, autonomic neuropathy *Recombinant human interferon-alpha:* Hypertension, hypotension, tachycardia, acute coronary events, dilated cardiomyopathy, arrhythmias, sudden death, atrioventricular block, peripheral vasodilation. Contraindicated in patients with unstable angina or recent myocardial infarction *Interleukin-2:* Hypotension, arrhythmia, sudden death, myocardial infarction, dilated cardiomyopathy, capillary leak, thyroid alterations *Anthracyclines (doxorubicin, daunorubicin, mitoxantrone):* Myocarditis, cardiomyopathy *Liposomal anthracyclines:* As above for doxorubicin and also vasculitis
Pentoxifylline		*Pentoxifylline:* Decreased triglyceride levels, arrhythmias, chest pain *Megace:* Edema, thrombophlebitis, hyperglycemia
Megestrol acetate (Megace)		*Epoetin alpha (erythropoietin):* Hypertension, ventricular dysfunction
Methadone		Prolonged QT interval
Amphetamines		Increased heart rate and blood pressure

Modified with permission from Fisher SD, Lipshultz SE. Chapter 72: Cardiovascular abnormalities in HIV-infected individuals. In: Braunwald's Heart Disease: A Textbook of Cardiovascular Medicine, Ninth Edition. Editors: Bonow RO, Mann DL, Zipes DP, Libby P. Philadelphia: Elsevier Saunders. 1618–27. 2011 ISBN: 978-1-4377-0398-6.

Mother-to-child transmission has been reduced in the United States to approximately 1–2% (CDC). Intrauterine exposures to these potent ARTs have been shown to have some effects on the child [118]. At birth, children exposed to HIV and ARTs were lighter than a comparison group with no exposures to ARTs and showed accelerated growth during the first two years of life. Additionally, these children had less subcutaneous fat and decreasing mid-upper arm circumference over time when compared to national standards [119].

### Perinatal transmission of HIV-infection

Although HIV transmission can be minimized if mothers are given ART in the second and third trimesters or short courses before parturition, most children with HIV are infected in the perinatal period [120]. Current therapies, some including up to six months of neonatal AZT, can limit the incidence of perinatal transmission to <2%. A worldwide UNAIDS goal is to eliminate perinatal transmission by the end of 2014.

Rates of congenital cardiovascular malformations ranged from 5.6 to 8.9% in cohorts of HIV-uninfected and HIV-infected children born to HIV-infected mothers. Although these rates were not higher than in similarly screened normal populations, they were 5 to 10 times as high as those reported in population-based epidemiological studies [120].

In the same cohorts, serial echocardiograms performed at four- to six-month intervals showed subclinical cardiac abnormalities to be common, persistent, and often progressive [5,62,68]. Some patients had dilated cardiomyopathy (LV contractility 2 standard deviations or more below the mean of a normative population and LV end-diastolic dimension 2 standard deviations or more above the mean) whereas others had mildly increased cardiac mass for height and weight. Depressed LV function correlated with immune dysfunction at baseline but not over time. This correlation suggests that the CD4 cell count may not be a useful surrogate marker of HIV-associated LV dysfunction. Disease can progress rapidly or slowly in children with perinatally-transmitted HIV-1 infection [62]. Rapid progressors have higher heart rates, higher respiratory rates, and lower fractional shortening on serial examinations than do non-rapid progressors and HIV-uninfected children who are similarly screened. Rapid progressors also have higher HIV-1 viral loads, higher five-year cumulative mortality, and lower CD8+ (cytotoxic) T-cell counts.

Studies of non-HIV-infected infants born to HIV-infected mothers have reported that foetal exposure to ART is associated with reduced LV dimension, LV mass, and septal wall thickness along with higher LV fractional shortening and contractility during the first two years of life [121]. *In utero* exposure to ART may initially improve LV function while impairing myocardial growth. Although LV function is improved, it is still below normal [68]. These effects are more pronounced in girls [68].

## Conclusions

### Cardiac monitoring recommendations

**Figure 5:**
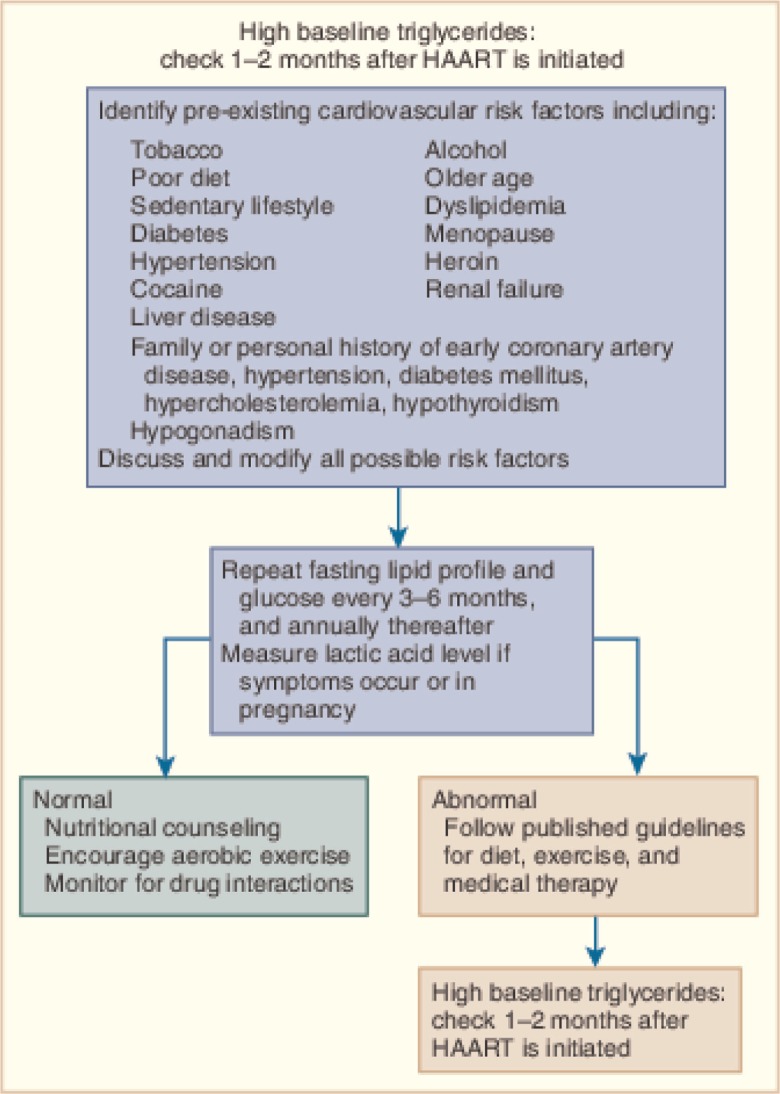
Cardiovascular considerations when initiating highly active antiretroviral therapy (HAART). Reproduced with permission from “Fisher SD, Lipshultz SE. Chapter 72: Cardiovascular abnormalities in HIV-infected individuals. In: Braunwald's Heart Disease: A Textbook of Cardiovascular Medicine, Ninth Edition. Editors: Bonow RO, Mann DL, Zipes DP, Libby P. Philadelphia: Elsevier Saunders. 1618–27. 2011 ISBN: 978-1-4377-0398-6.”

Routine, systematic cardiac evaluation, including a comprehensive history and thorough cardiac examination, is essential care for HIV-infected children and adults. The history should include traditional risk factors, environmental exposures, prior opportunistic infections, and therapeutic and illicit drug use. Laboratories should include a lipid profile, fasting glucose, and HIV viral load (Figure 5). Routine blood pressure monitoring is important because HIV-infected individuals can experience hypertension at a younger age and more frequently than in the general population [20,33,52].

Unless patients have symptoms such as palpitations, syncope, stroke, or dysautonomia, routine ECG and Holter monitoring are not warranted. These tests can be useful for baseline and monitoring before, during, and after therapies, such as pentamidine, methadone, or antibiotics that may prolong the QT interval [108].

Asymptomatic cardiac disease related to HIV can be fatal. When present, cardiac symptoms are often disguised by secondary effects of HIV infection. Thus, systematic echocardiographic monitoring is warranted [64,65,122,123]. An international consensus panel recommended echocardiographic monitoring, with a baseline, for any patient at high risk or with any clinical manifestation of CVD, in addition to studies every 1–2 years or as clinically indicated. Patients with cardiac symptoms should begin directed therapy and receive a formal cardiac assessment, including baseline ECG, echocardiography, and Holter monitoring [124]. Brain natriuretic peptide concentrations may help diagnose ventricular dysfunction [125,126].

Serum troponin assays are indicated in patients with LV dysfunction. Elevated concentrations of serum troponin warrant consideration of endomyocardial biopsy and cardiac catheterization. Therapy with intravenous immunoglobulin should be considered for biopsy-proven myocarditis [2]. Echocardiography should be repeated after two weeks of therapy to encourage continued therapy if improvement has occurred and adapt a more aggressive approach if LV dysfunction persists or worsens.

Stress testing and coronary assessment such as CT angiography or cardiac catheterization should be considered in the appropriate clinical settings [33,44,54,62]. Guidelines for using implantable cardioverter-defibrillators should be followed in this population, especially in patients after MI being treated for HIV infection [33].

As a chronic disease, HIV-related CVD is a vital area of research. If HIV can be used as a model of chronic immunosuppression in a large population, findings may translate to other populations. Understanding genetic predispositions to QT prolongation may guide therapy. Understanding the causes of cardiomyopathy may benefit diverse research efforts, such as the effects of cytokines, mitochondria, and neurohormonal pathways. Observations, such as increased mortality related to LV mass and very mild LV dysfunction might enhance diagnostic testing in at-risk populations affected by other poorly understood cardiomyopathies.
